# Mastering Interproximal Stripping: With Innovations in Slenderization

**DOI:** 10.5005/jp-journals-10005-1159

**Published:** 2012-08-08

**Authors:** Narendra Shriram Sharma, Sunita S Shrivastav, Pushpa V Hazarey

**Affiliations:** Assistant Professor, Department of Orthodontics, Sharad Pawar Dental College, Sawangi, Wardha, Maharashtra, India, e-mail: sharmanarendra047@gmail.com; Professor, Department of Orthodontics, Sharad Pawar Dental College Wardha, Maharashtra, India; Professor and Head, Department of Orthodontics, Sharad Pawar Dental College, Wardha, Maharashtra, India

**Keywords:** Minor crowding, Slenderization, Preventive Orthodontic

## Abstract

Crowding and irregularity remain a consistent problem for children. Management of space problems continues to play an important role in a dental practice. It also represents an area of major interaction between the primary provider and the specialists. Proximal stripping is routinely carried out to avoid extraction in borderline cases where space discrepancy is less and in cases where there is a discrepancy between the mesio- distal width of maxillary and mandibular teeth to satisfy Bolton ratio. Proximal stripping is carried out using of metallic abrasive strip, safe sided carborundum disk, or with long thin tapered fissure burs with air rotor.

The use of rotary cutting instrument can harm the pulp by exposure of mechanical vibration and heat generation (in some cases). Whereas, the large diameter of the disk obstructs vision of the working area. Also fracturing away a portion is a common problem with disk. Tapered fissure burs cut the tooth structure as the width of bur or overcutting may occur of the tooth structure due to high speed. The use of metallic abrasive strip is the safest procedure amongst the above. The strip can be placed in the anterior region without any difficulty but using it in the posterior region is difficult as, it is difficult to hold it with fingers while stripping the posterior teeth.

To avoid this inconvenience here with a simple and economical way of fabricating strip holder from routine lab material is presented.

**Clinical implications:** Proper management of space in the primary and mixed dentitions can prevent unnecessary loss in arch length. Diagnosing and treating space problems requires an understanding of the etiology of crowding and the development of the dentition to render treatment for the mild, moderate and severe crowding cases. Most crowding problems with less than 4.5 mm can be resolved through preservation of the leeway space, regaining space or limited expansion in the late mixed dentition. In cases with 5 to 9 mm of crowding, some can be approached with expansion after thorough diagnosis and treatment planning. Most of these cases will require extraction of permanent teeth to preserve facial esthetics and the integrity of the supporting soft tissue. Sequential proximal stripping is routinely carried out to avoid extraction in borderline cases where space discrepancy is less and reserved for treatment of mild tooth-size/arch-size discrepancies.

**How to cite this article:** Sharma NS, Shrivastav SS, Hazarey PV. Mastering Interproximal Stripping: With Innovations in Slenderization. Int J Clin Pediatr Dent 2012;5(2):163-166.

## INTRODUCTION

Crowding and irregularity remain a consistent problem for children. Management of space problems continues to play an important role in a dental practice. It also represents an area of major interaction between the primary provider and the specialists. Proximal stripping is routinely carried out to avoid extraction in borderline cases where space discrepancy is less and in cases where there is a discrepancy between the mesiodistal width of maxillary and mandibular teeth to satisfy Bolton ratio. Creating space in order to facilitate tooth movement is one of the basic principles of orthodontics. If space is required then expansion, extraction, or tooth slenderization must be performed. As patients seek faster orthodontic treatment, extraction is becoming reserved for cases in which severe crowding is present, where there is a need for vertical change or control, or where sagittal correction/compensation cannot be accomplished without extraction. Sequential proximal stripping is routinely carried out to avoid extraction in borderline cases where space discrepancy is less and reserved for treatment of mild tooth- size/arch-size discrepancies.

### The Basic History of Slenderization

In 1902, Black^1^ published a text on tooth anatomy that discussed the natural interproximal abrasion of teeth (natural slenderization). In 1944, Ballard^2^ described the slenderization technique for the first time. Both Sheridan^3^ in labial technique, and Fillión^4^ in lingual technique, among others, have contributed to the development of the slenderization technique currently in use. Studies by Begg^5^ and Murphy^6^ on the occlusions of Aboriginals found that they presented with interproximal wear, amounting to the loss of up to 14 to 15 mm of dental material during a lifetime as a consequence of nonrefined diets and the absence of crowding. Sicher,^7^ speaking about tooth attrition, stated that it was possible that tooth wear has a positive function and asked whether nature sacrifices tooth substance to achieve an increase in functional potentiality. Peck and Peck^8^ found a relationship between dental size (mesiodistal and labiolingual distances of the inferior incisors) and crowding grade (PI index). Betteridge^9^ also found a relationship between dental size and crowding grade (BI index).

Teeth vary in size between females and males, mostly in the permanent dentition. Males' teeth are larger than those of females,^10-15^ with maxillary centrals and canines showing the greatest differences.^15^ Bolton^16^ analyzed the relationships between canine-to-canine widths and molar-to-molar widths in dental arches, and found tooth size discrepancies in approximately 30% of his patients. Freeman, Santoro and Alexander^17^ also observed similar percentages in their studies. Sassouni^18^ pointed out that class III facial types and patients with deûcient maxillary growth show a greater incidence of anterior tooth shapes and agenesis. Cua-Benward^19^ found similar results in class III subjects, and tooth deformities in the lower anterior region in class II individuals.

### Indications for Slenderization

Slenderization is indicated when the treatment goals require space in the dental arches without the removal of teeth. It is also indicated in cases where individual tooth sizes prevent a class I molar and canine relationship.

### Contraindications for Tooth Slenderization

Generally speaking, slenderization should be avoided on: Small teeth, restored teeth possessing a normal shape, teeth with enamel hypoplasia, and severely rotated teeth for which access to the proper contact area is not accessible (in cases like this, it is recommended to either make room using the separation technique or wait until crowding in the area of the tooth is resolved and space is created). It should also be avoided in patients who refuse to accept slenderization as a treatment option (informed consent is imperative); patients with high caries index, poor hygiene, high bacterial plaque index, or rectangular-shaped teeth; and young patients with large pulp chambers.

### Advantages of Slenderization

Slenderization minimizes potential consequences created by extraction, which can include:

- Difficulties in complete space closure.
- Difficulties in paralleling the roots next to extraction sites.
- Need for a greater anchorage reinforcement than in slenderization cases (but the anchorage is fundamental in the slenderization technique, too).
- Possibility of the space reopening (relapse), especially in adult patients.
- Unwanted profile changes related to retroclining incisors when closing extraction spaces.

When slenderizing, dental movements are smaller than in extraction cases. The slenderization treatments are shorter. The risk of root resorption is also reduced. Some orthodontists believe that contact points between teeth flattened during slenderization are more stable for rotation control, eliminating the relapse risk. Slenderization allows the ‘black gingival triangles' to be avoided or reduced, dental asymmetries to be compensated and when needed, dental shape to be improved.

### Disadvantages of Slenderization

Techniques which do not emphasize conservativeness, along with operator error, can result in enamel damage or over- reduction. Contours of teeth can easily be destroyed, after which a restorative procedure is required. Performing slenderization with instruments with which the operator can lose control of the procedure, such as ARS (air rotor stripping), is not recommended. This can result in spacing that requires subsequent orthodontic treatment for closure. High-speed spinning diamond disks easily slice teeth, as the disk takes its own path while spinning, and are not recommended. To control the reduction of tooth structure, a low-speed, high-torque handpiece should be used.

### Instruments Used to Slenderize

- Stainless steel strips
- Manual disk hand tool
- High-torque diamond disks
- ARS (air rotor slenderization) burs and diamond disks.

### Procedure

**Fig. 1:**
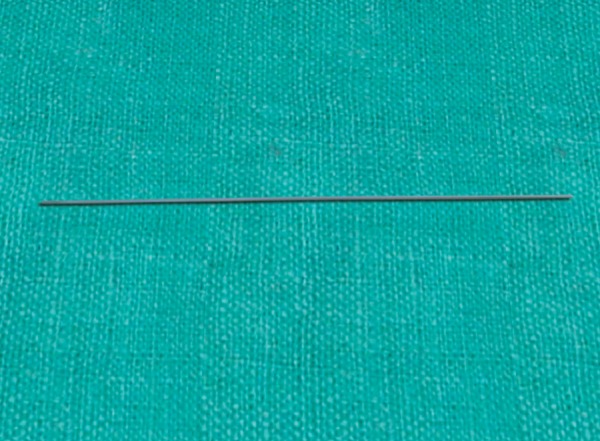
Take a 10 inch length of 0.32" or 0.36" stainless steel wire

**Fig. 2:**
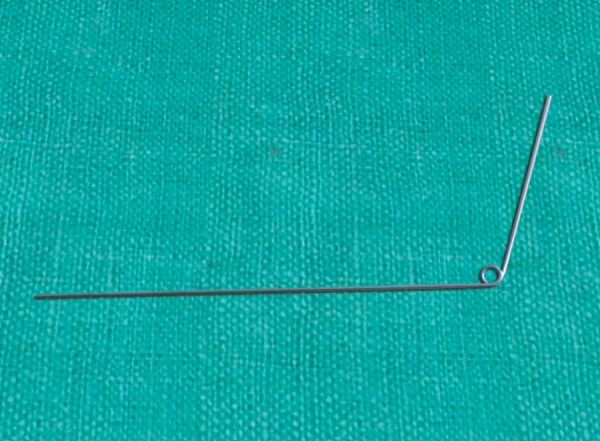
Make a 4 mm radius helix at a 4 inch distance from one end of wire

**Fig. 3:**
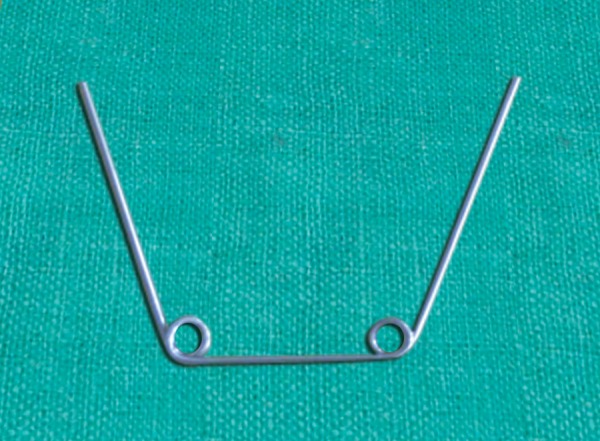
Repeat the helix at 1 inch distance form first helix on other side of the wire

**Fig. 4:**
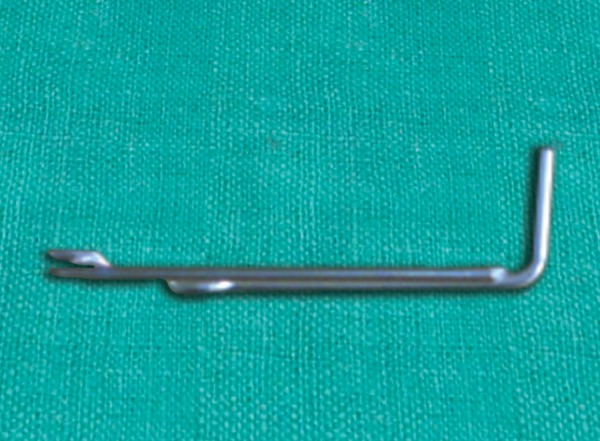
Give 90% horizontal bend in vertical arm of free side of wire

**Fig. 5:**
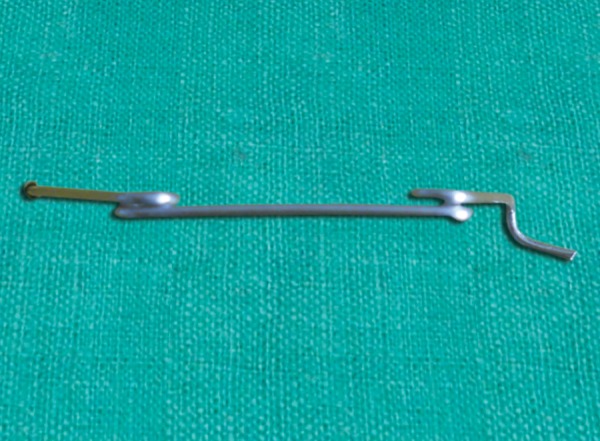
Curve the wire 2 mm away from 90° bend and cut the excess wire

**Fig. 6:**
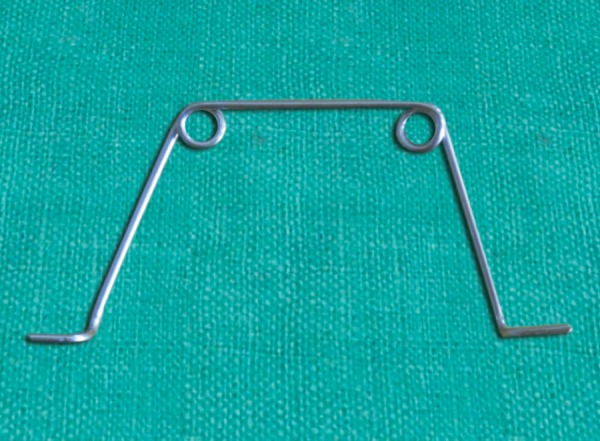
Repeat the same bend in vertical arm of wire below helix

**Fig. 7:**
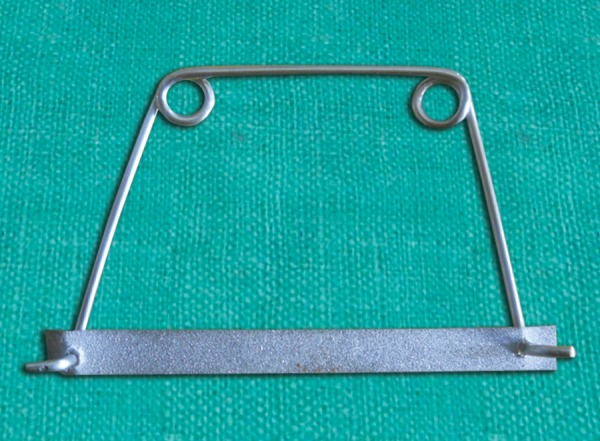
Take a strip of approximate length, punch a hole in it with dental probe and placed it on holder

**Figs 8A and B:**
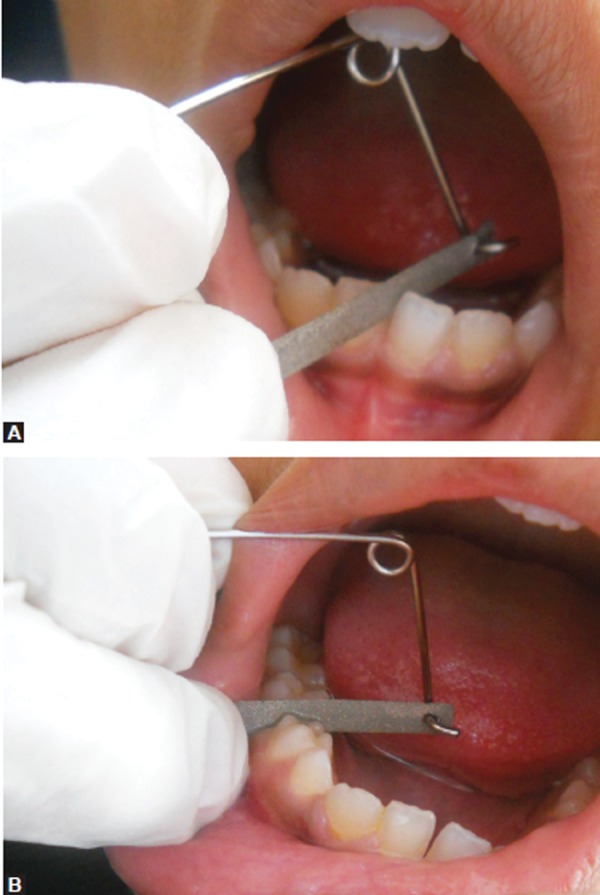
Place a strip in the desired interdental region to cut the tooth material

1. Take a 10 inch length of 0.32" or 0.36" stainless steel wire (Fig. 1).
2. Make a 4 mm radius helix at a 4 inch distance from one end of wire (Fig. 2).
3. Repeat the helix at 1 inch distance form first helix on other side of the wire (Fig. 3).
4. Give 90% horizontal bend in vertical arm of free side of wire (Fig. 4).
5. Curve the wire 2 mm away from 90° bend and cut the excess wire (Fig. 5).
6. Repeat the same bend in vertical arm of wire below helix (Fig. 6).
7. Take a strip of approximate length, punch a hole in it with dental probe and placed it on holder (Fig. 7).
8. Place a strip in the desired interdental region to cut the tooth material (Figs 8A and B).

### Advantages

1. Easy insert and removal.
2. The strip remains tight irrespective of span of wire used, as helix can be adjusted.
3. Can be sterilize.
4. It can be fabricated chairside in minimum time.
5. A single sided strip also can be used.
6. No stress is incorporated in wire during fabrication.
7. Type of handle which is available commercially is costly and there is excess strip loss. The amount of strip used by this technique is cost-effective.

### What this Paper Adds?

- Prevantive orthodontics
- Diagnosing and treating space problems
- Proper management of space
- If the case is properly diagnosed then slenderization is safe, easy to apply and well-accepted by patients. This shows that it may offer an efficient, nonsurgical alternative for the treatment of borderline cases where space discrepancy is less.
- Most of the time proper management of space in the primary and mixed dentitions can prevent unnecessary loss in arch length. A patient in younger age is screen by pedodontist. It is important to diagnose a case properly so, that sequential proximal stripping is carried out to avoid extraction in borderline cases where space discrepancy is less and reserved for treatment of mild tooth-size/arch-size discrepancies.
- This procedure can be easily performed by pedodontist in his office.
- No special skill is required.
- No special armamentarium is required.

## References

[1] Black GV (1902). Descriptive anatomy of the human teeth..

[2] Ballard R, Sheridan JJ, (Louisiana State University School of Dentistry, New Orleans 70119, USA) (1996). Air-rotor stripping with the Essix anterior anchor.. J Clin Orthod.

[3] Sheridan JJ (1985). Air-rotor stripping.. J Clin Orthod.

[4] Fillion D. (1993). Apport de la sculpture amálaire interproximale àl'ortodontie de l'adulte (troisième partie).. Rev Orthop Dento Faciale.

[5] Begg PR. (1965). Begg orthodontic theory and technique..

[6] Murphy TR (1964). Reduction of the dental arch by approximal attrition.. Br Dent J.

[7] Sicher H (1953). The biology of attrition.. Oral Surg Oral Med Oral Pathol.

[8] Peck H, Peck S (1972). An index for assessing tooth shape deviations as applied to the mandibular incisors.. Am J Orthod.

[9] Betteridge MA (1976). Index for measurement for lower labial segment crowding.. Br J Orthod.

[10] Garn SM, Lewis AB, Kerewsky RS (1964). Sex difference in tooth size.. J Dent Res.

[11] Beresford JS (1969). Tooth size and class distinction.. Dent Pract Dent Rec.

[12] Sanin C, Savara BS (1971). An analysis of permanent mesiodistal crown size.. Am J Orthod.

[13] Potter RH (1972). Univariate versus multivariate differences in tooth size according to sex.. J Dent Res.

[14] Arya BS, Savara BS, Thomas D, Clarkson Q (1974). Relation of sex and occlusion to mesiodistal tooth size.. Am J Orthod.

[15] Doris JM, Bernard BW, Kuftinec MM, Stom D (1981). A biometric study of tooth size and dental crowding.. Am J Orthod.

[16] Bolton WA (1958). Disharmony in tooth size and its relation to the analysis and treatment of malocclusion.. Angle Orthod.

[17] Alexander RG, Engel GA. (1986). The Alexander discipline contemporary concepts and philosophies.. Ormco Corporation.

[18] Sassouni V (1969). A classification of skeletal facial types.. Am J Orthod.

[19] Cua-Benward GB, Dibaj S, Ghassemi B, (Harvard School of Dental Medicine, Boston, Massachusetts) (1992). The prevalence of congenitally missing teeth in class I, II and III malocclusions.. J Clin Pediatr Dent.

